# Disentangling Vector-Borne Transmission Networks: A Universal DNA Barcoding Method to Identify Vertebrate Hosts from Arthropod Bloodmeals

**DOI:** 10.1371/journal.pone.0007092

**Published:** 2009-09-21

**Authors:** Miguel Alcaide, Ciro Rico, Santiago Ruiz, Ramón Soriguer, Joaquín Muñoz, Jordi Figuerola

**Affiliations:** 1 Estación Biológica de Doñana (CSIC), Isla de la Cartuja, Sevilla, Spain; 2 Diputación de Huelva, Área de Medio Ambiente, Huelva, Spain; BMSI-A*STAR, Singapore

## Abstract

Emerging infectious diseases represent a challenge for global economies and public health. About one fourth of the last pandemics have been originated by the spread of vector-borne pathogens. In this sense, the advent of modern molecular techniques has enhanced our capabilities to understand vector-host interactions and disease ecology. However, host identification protocols have poorly profited of international DNA barcoding initiatives and/or have focused exclusively on a limited array of vector species. Therefore, ascertaining the potential afforded by DNA barcoding tools in other vector-host systems of human and veterinary importance would represent a major advance in tracking pathogen life cycles and hosts. Here, we show the applicability of a novel and efficient molecular method for the identification of the vertebrate host's DNA contained in the midgut of blood-feeding arthropods. To this end, we designed a eukaryote-universal forward primer and a vertebrate-specific reverse primer to selectively amplify 758 base pairs (bp) of the vertebrate mitochondrial Cytochrome *c* Oxidase Subunit I (COI) gene. Our method was validated using both extensive sequence surveys from the public domain and Polymerase Chain Reaction (PCR) experiments carried out over specimens from different Classes of vertebrates (Mammalia, Aves, Reptilia and Amphibia) and invertebrate ectoparasites (Arachnida and Insecta). The analysis of mosquito, culicoid, phlebotomie, sucking bugs, and tick bloodmeals revealed up to 40 vertebrate hosts, including 23 avian, 16 mammalian and one reptilian species. Importantly, the inspection and analysis of direct sequencing electropherograms also assisted the resolving of mixed bloodmeals. We therefore provide a universal and high-throughput diagnostic tool for the study of the ecology of haematophagous invertebrates in relation to their vertebrate hosts. Such information is crucial to support the efficient management of initiatives aimed at reducing epidemiologic risks of arthropod vector-borne pathogens, a priority for public health.

## Introduction

The control of emerging infectious diseases constitutes one of the most important concerns of global economies and human health. Recent studies have emphasized that the majority of the last pandemics have been originated by zooneses in the wild. The participation of vectors in the spread of zoonotic diseases was estimated to occur in about one fourth of pathogen outbreaks during the last century [1]. Importantly, human related activities are contributing to increase the impact of vector-borne diseases by increasing vector density (e.g. irrigation, urbanization, dam construction) or by introducing pathogens into areas in which they had been hitherto absent [2]. As a result, there has been the need to control the population of blood-feeding arthropods to reduce epidemiologic risks because they may harbor pathogens responsible for serious infectious diseases such as malaria, viral encephalitis, West Nile virus, Chagas disease, Lyme disease or African sleeping sickness. Although pathogen prevalence in vectors is usually low, a correct understanding of vector-hosts interactions is crucial to predict transmission patterns and for the development of efficient control policies.

The enhancement of species identification since the advent of molecular methods has revolutionized our view of such complex ecological networks at the same time that it has contributed to gain insights on the co-evolutionary relationships between vectors, pathogens and their hosts [reviewed in 3]. The emerging of international initiatives such as the Consortium for the Barcode of Life (www.barcoding.si.edu) is expected to greatly expand the benefits of molecular methods in this field. The DNA barcoding project aims to establish a 648-bp fragment of the Cytochrome *c* Oxidase Subunit I (COI) mitochondrial gene as universal method for the taxonomic classification of biodiversity [4], [5]. The coverage provided by barcoding databases will soon facilitate an accurate assignment of host-vector associations, and their use by ecologists and epidemiologists is believed to increase considerably during the next few years [6]. One of the most important advantages of the COI locus to identify vertebrate hosts is linked to its model of molecular evolution, which is believed to provide better resolution of deeper taxonomic affinities than other molecular markers [7], [8]. In this sense, precise host identifications might be crucial given that pathogen outcomes, exposure to vectors, demographic parameters and dispersal patterns may vary considerably even between closely related species [9]–[11]. Nevertheless, identification methods based on the partial sequencing of the vertebrate COI gene from arthropod bloodmeals have been underutilized in relation to other molecular approaches [3]. In fact, COI identification protocols conducted to date have focused exclusively on mosquito bloodmeals [3], [12]. There is therefore the need to ascertain and take advantage of the potential afforded by DNA barcoding tools in other vector-host systems of public health and veterinary importance.

In this study, we have designed a single pair of primers for the selective analysis of host COI sequences from arthropod bloodmeals. Our method was also aimed at accelerating host identification and reducing both laboratory efforts and costs.

## Results

### Validation of vertebrate-specific PCR amplification

Primers M13BC-FW and BCV-RV1 were validated using high quality DNA extracts from avian (lesser kestrel *Falco naumanni*, Spanish Imperial eagle *Aquila adalberti*), mammalian (water vole *Arvicola sapinus*), amphibian (natterjack toad *Bufo calamita*) and reptilian (Iberian wall lizard *Podarcis hispanica*) species. PCR experiments always yielded high concentration of amplicons of the expected size. The same PCR protocol failed to amplify the target fragment when applied to DNA extracts obtained from the abdomen of non-engorged arthropods (mosquitoes, ticks, culicoids and sandflies). Nonetheless, we successfully amplified and sequenced a fragment of the arthropod COI locus using the eukaryote-universal primers proposed for the ‘DNA mini-barcoding’ approach [13]. It is important to emphasize that eukaryote-universal primers preferentially amplified vector COI sequences when applied to mosquito bloodmeals. On the contrary, our vertebrate-specific primer set only replicated host COI genes.

### Assignment of unknown COI sequences replicated from bloodmeals to specific vertebrate hosts

The first PCR reaction with primers M13BC-FW and BCV-RV1 yielded suitable concentrations of PCR products for sequencing in 43 out of 100 mosquito bloodmeals. Non-suitable samples for sequencing displayed either low concentration of PCR products or a lack of bands of the expected size. Nonetheless, a nested PCR using M13 and BCV-RV2 primers generated suitable positives for sequencing in 97 out of 100 mosquito bloodmeals. The nested-PCR also proved satisfactory when applied to the bloodmeals of additional vector species (ticks, sandflies, culicoids and blood-sucking bugs).

The bioinformatics platform supported by the Barcode of Life Data Systems (BOLD) database permitted us to identify the origin of the bloodmeals contained in the midgut of several species of mosquitoes and other blood-fed ectoparasites (see Table 1). This is an online workbench that aids collection, management, analysis, and use of DNA barcodes by researches in different fields (www.barcodinglife.org). The inspection of sequencing electropherograms supported the amplification of the mitochondrial barcode locus from one single vertebrate host in the vast majority of cases. The similarity of our unknown vertebrate COI sequences with respect to those from museum voucher specimens was always >99% except for five cases. Various unknown vertebrate COI sequences could not be assigned at the species level. These COI sequences showed the highest sequence similarity (>90%) with species of the genus *Lepus*, *Lynx*, *Mus*, *Alectoris* and the Family *Herpestidae*. Given that the unique members of the genus *Lepus*, *Alectoris* and the Family *Herpestidae* inhabiting the south of Spain are not included within the BOLD System database yet, we deduced that these species could be the Iberian hare *Lepus granatensis*, the red-legged partridge *Alectoris rufa* and the Egyptian mongoose *Herpestes ichneumon*. Furthermore, we know that the two remaining COI sequences belonged to the Iberian Lynx *Lynx pardinus* and the Algerian mouse *Mus spretus* because ectoparasites were directly sampled on hosts. Overall, our molecular method allowed identifying 40 different vertebrate hosts, including 16 mammalian, 23 avian and one reptilian species (see Table 1). DNA extraction and PCR negative controls did not yield PCR bands and the analysis of our positive control always matched COI sequences of the mallard duck *Anas platyrhynchos*. Repeatability experiments performed over those bloodmeals reporting rare hosts (i.e. those found only in one bloodmeal) were also successful.

**Table 1:** Vertebrate hosts for different species of blood-feeding ectoparasites collected in South-western Spain.

Species	Mammalian host	Avian host
Anopheles algeriensis (Insecta: Culicidae)	Dama dama (1); Bos taurus (1)	
Anopheles atroparvus (Insecta: Culicidae)	Bos taurus (3); Oryctolagus cuniculus (1)	
Culex modestus (Insecta: Culicidae)		Anas platyrhynchos (2); Anser anser (1); Chen caerulescens (1); Branta canadensis (1); Egretta garzetta (1); Tadorna ferruginea (1); Anas strepera (1); Gallus gallus (1); Ardea cinerea (1); Anas acuta (1); Tadorna tadorna
Culex perexiguus (Insecta: Culicidae)	Rattus norvergicus (1); Canis familiaris (1)	Alectoris rufa (1); Streptopelia decaocto (1)
Culex pipiens (Insecta: Culicidae)	Homo sapiens (1); Herpestes ichneumon (1); Felis catus (1); Canis familiaris (2)	Passer domesticus (7); Turdus merula (3); Streptopelia decaocto (3); Galerida cristata (1); Sturnus vulgaris (2); Cairina moschata (1); Grus grus (1); Sylvia melanocephala (1); Alectoris rufa (1)
Culex theileri (Insecta: Culicidae)	Bos taurus (8); Cervus elaphus (4); Dama dama (2); Equus caballus (3); Homo sapiens (1); Lepus granatensis (2); Oryctola gus cuniculus (1); Sus scrofa (2)	Bubulcus ibis (1); Meleagris gallopavo (1)
Ochlerotatus caspius (Insecta: Culicidae)	Bos taurus (3); Canis familiaris (3); Capra hircus (1); Felis catus (1); Cervus elaphus (1); Equus caballus (2); Oryctolagus cuniculus (2); Ovis aries (2); Sus scrofa (1)	Gallus gallus (4); Podiceps nigricollis (1); Passer domesticus (2); Turdus merula (1); Sturnus vulgaris (1)
Culiseta longiareolata (Insecta: Culicidae)		Passer domesticus (2)
Phlebotomus sp. (Insecta: Phlebotominae)	Oryctolagus cuniculus (2)	
Culicoides sp. (Insecta: Ceratopogonidae)		Passer domesticus (2)
Dipetalogaster maximus (Insecta: Reduviidae)	Lynx pardinus (2)	
Rhipicephalus spp. (Arachnida: Ixodidae)	Mus spretus (1) Canis familiaris (2)	

We isolated either mammalian or avian DNA from arthropod bloodmeals, except for a *Culex modestus* mosquito that fed on a turtle (*Mauremys leprosa*, not included because of limitations in table format). Brackets indicate the number of bloodmeals reporting DNA from particular hosts in each vector species.

### Identification of mixed bloodmeals

**Figure 1:**
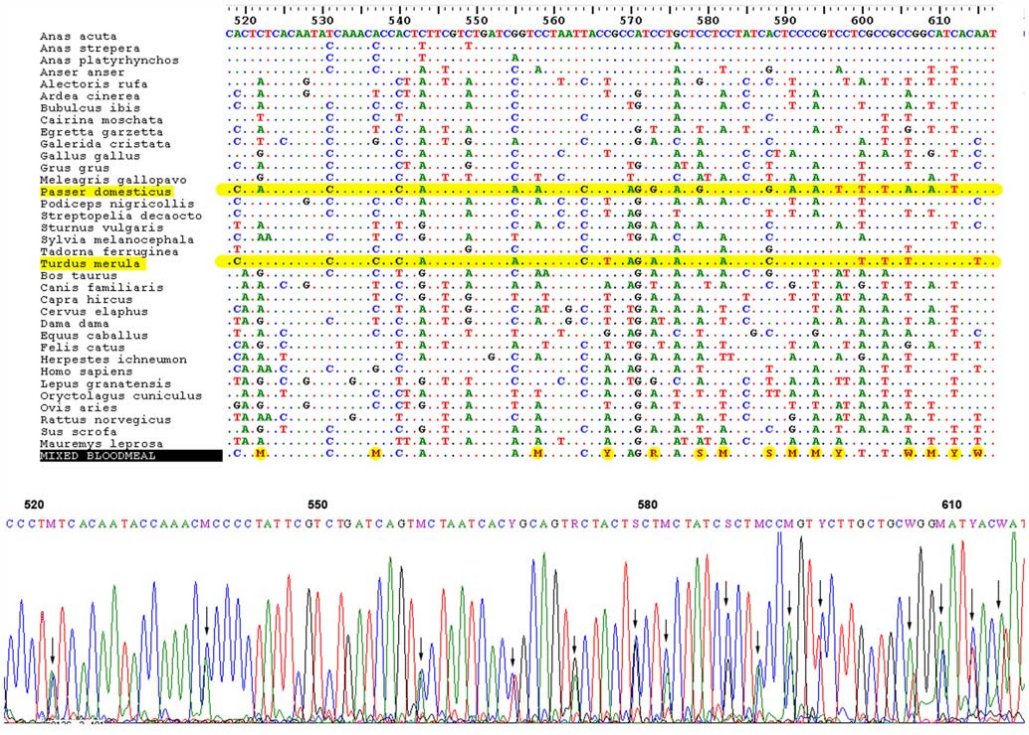
Identification of a mixed bloodmeal. The co-amplification of COI fragments from different hosts may generate several ambiguous nucleotide positions (in yellow and indicated by arrows). The ambiguous sequence can be subsequently compared with respect to a validated set of COI sequences from the hosts typically found in the study area. In this example, the sequencing electropherogram obtained could be the result of the simultaneous amplification of DNA from house sparrow *Passer domesticus* and common blackbird *Turdus merula*.

The alignment of ambiguous COI sequences (i.e. those displaying double peaks at different positions of sequencing electropherograms) with respect to our validated set of vertebrate COI sequences indicated four mixed bloodmeals. The inspection of sequencing electropherograms suggested the co-amplification of the COI locus from two different vertebrate hosts. A specimen of *Ochlerotatus caspius* and a specimen of *Culex pipiens* could have fed on a house sparrow *Passer domesticus* and a common blackbird *Turdus merula* (see Fig. 1). A specimen of *Culex modestus* could have fed on a common goose *Anser anser* and a domestic snow goose *Chen caerulescens*, and a specimen of *Culex theileri* could have fed on a red deer *Cervus elaphus* and a cow *Bos taurus*. In some cases, particularly for the wild boar *Sus scrofa*, we suspected that double peaks could be related to the co-amplification of nuclear insertions of the mitochondrial genome or heteroplasmy. We submitted unambiguous stretches of COI sequences to the public databases to ensure that these DNA sequences could only be originated from the same species. Then, sequence identities larger than 99% were only reported with respect to the same, single vertebrate host.

## Discussion

In this study, we describe a straightforward and universal method for the PCR specific amplification and analysis of the vertebrate barcode COI locus from the midgut of blood-feeding arthropods. The suitability of a single pair of primers capable to cope with all candidate hosts across a broad spectrum of vectors constitutes the major improvement put forward by our method. Although single pairs of primers were formerly available for the amplification of the vertebrate COI [12] and Cytochrome *b* (Cyt *b*) genes [14], empirical support for their suitability in other vector species rather than mosquitoes and even the possible co-amplification of invertebrate DNA, respectively, could be considered as major limitations. In fact, the vast majority of studies have employed cocktails of primers to deal with different vertebrate hosts [reviewed in 3]. The inconvenience of using avian or mammalian specific primers for those vectors traditionally considered ornithophilic or mammalphilic is also highlighted in the present study. As an example, we found that some mosquito species such as *Culex pipiens*, *Cx. theileri* and *Ochlerotatus caspius* can feed occasionally on other vertebrates groups (see Table 1). Shedding light on this kind of relationships would provide valuable information to understand pathogen transmission between different vertebrate groups.

One of the most interesting methodological advantages of our assay relies on the suitability of the inexpensive HotSHOT protocol for a rapid and feasible DNA extraction from bloodmeals. Success of our nested-PCR approach was >90% for the bloodmeals analyzed, a similar value to other studies based on the amplification of a shorter fragment (<400 bp) of the Cyt *b* gene [e.g. 15]. Assuming a Taq DNA Polymerase error rate about 1×10^−4^ base substitutions/bp/cycle, the total number of mutations introduced by our nested PCR protocol is around 8.24 base substitution per sequence (i.e. 1×10^−4^×758 bp×110 cycles (75 PCR cycles +35 Big Dye cycles)). This estimate represents 1% of overall nucleotide divergence and will not exceed in any case the 2% threshold of within-species variation established by the BOLD initiative. PCR costs and sample manipulation can be considerably reduced if the number of engorged arthropods is large enough to make unnecessary the analysis of those specimens containing tiny bloodmeals or bloodmeals into an advanced digestion stage. Direct sequencing methods, which represent >80% of overall costs, can be also replaced by other less expensive and more straightforward molecular methods [reviewed in 3] once researchers have accomplished a first preliminary survey using our general approach. The application of our method throughout hotspots of biodiversity is however strongly encouraged given that alternative methods can difficultly provide similar coverage thresholds and sample manipulation convenience.

Importantly, we show that the analysis of sequencing electropherograms could be useful for the identification of mixed bloodmeals from even closely related species (see Fig. 1). The development of specific software to assist the identification of mixed sequencing electropherograms using the sequence data deposited in the public databases must be encouraged in this respect. PCR reactions can be nevertheless highly competitive in mixed bloodmeals and some DNA data could be missing due to partial or total degradation of one of the host's DNA and/or because of the amount of ingested blood from different host species greatly differs. To solve this potential methodological limitation, some authors have satisfactorily used multiplexed primers targeting to different vertebrate groups [16], [17], [but see the multiplexing drawbacks reviewed in 3]. In the very next future, new generation and cost-effective sequencing technologies such as pyrosequencing are expected to revolutionize bloodmeal analyses because it will permit researchers to simultaneously screen a wider array of DNA sequences from mixed samples at different proportions [18], [19].

Our method could have a limited value, however, for those species parasitizing known hosts most of the time (e.g. ticks, fleas, mites or lices). Engorged ectoparasites can be nonetheless collected off-hosts using traps or drag sampling [20], [21]. Ticks deserve a particular consideration because of their mechanism of blood digestion differs from the rest of blood-feeding arthropods. DNA digestion seems to occur more quickly, and PCR-based methods have proven to vary considerably between different laboratories. Thus, recent research has encouraged the use of tryptic-digestion mass spectrometry of blood proteins to identify various hosts with a single tick bloodmeal [22]. Nevertheless, increased costs and a very limited availability of protein databases for non-model species are two important drawbacks.

In conclusion, we provide a novel, relatively straightforward and cost-effective molecular method that permits researchers to get deeper into the investigation of vector-borne disease ecology and co-evolutionary relationships. The reinforcement of our knowledge about blood-feeding behaviour of haematophagous arthropods and transmission patterns in wild species should decisively contribute to an efficient evaluation and modelling of epidemiologic risks and a better understanding of ecological networks.

## Materials and Methods

### Sampling of engorged ectoparasites and study area

Wildlife engorged mosquitoes, culicoids and sand flies (Class *Insecta*, Order Diptera, Families *Culicidae*, *Ceratopogonidae* and *Phlebotomiae*) were captured using CDC traps supplied with dry ice to attract ectoparasites through light and CO_2_. Traps were placed in several locations of South-western Spain, including the Doñana National Park, and the specimens collected the next day. This fact is important given that some studies have shown that the probability of amplifying host DNA after 36 hours from feeding decreases considerably [23]. Engorged ticks (Class *Arachnida*, Order Acari) were directly sampled from known parasitized hosts (mice and dogs). Individual ectoparasites were identified at the species or genus level. In addition, pathogen-free sucking bugs *(Dipetalogaster maximus*, Class *Insecta*, Order Hemiptera, Subfamily *Triatominae*) used for non-invasive blood sampling [see 24] of captive Iberian lynxs *Lynx pardinus* (www.lynxexsitu.es), were also obtained. Ectoparasites were preserved at −80°C until DNA extraction. Overall, we sampled 100 engorged mosquitoes plus a few specimens from other families of blood-feeding ectoparasites (Table 1).

### DNA extraction

Individual ectoparasites were processed in Petri plates. Using sterile tips, we pressed deeply on the abdomen of engorged individuals to release bloodmeals. According to the HotSHOT protocol [25], bloodmeals were pipetted into 50 µl of lysis solution (25 mM NaOH, 0.2 mM EDTA) and latter incubated at 95°C during 30 min. In those cases where bloodmeals could not be easily extracted from the midgut of the ectoparasite, we cut the entire abdomen, which was introduced and crushed into the lysis solution. In addition, at least two negative DNA extraction control (i.e. absence of tissue) were performed during PCR experiments. After incubation, the solution was put on ice for five minutes and then we added 50 µl of neutralization solution (40 mM Tris-HCl). Bloodmeals or abdomens were simultaneously processed using 96-thermowell plates or 8-thermowell individual strips and stored at −20°C until PCR amplification.

### Primer design strategy

We downloaded all vertebrate COI sequences (N = 18,928) from the Classes *Mammalia*, *Aves*, *Amphibia* and *Reptilia* that were available in the public domain managed by the BOLD Systems database in January 2009 (www.barcodinglife.org). We also downloaded 6,784 arthropod COI sequences from taxonomic groups that included blood-feeding species. This survey included species from the Classes *Arachnida* (Order Acari) and *Insecta* (Order Diptera, Families *Culicidae*, *Simuliidae*, *Ceratopogonidae*, *Hippoboscidae*, *Tabanidae*and *Glossinidae*; Order Hemiptera, Subfamiliy*Triatominae* and Order Phthiraptera). COI sequences were aligned using the software BioEdit 7.0.9.0 [26]. Our multiple alignments revealed several conserved nucleotide positions at the 5′ end of the COI gene. We designed a universal-forward primer with an M13-tail added at the 5′ end (M13BC-FW 5′-TGT AAA ACG ACG GCC AGT-HAA YCA YAA RGA YAT YGG NAC-3′), similar to other primers previously used in other barcode approaches [e.g. 13]. Then, we searched for a reverse primer that allowed the specific amplification of vertebrate COI sequences while avoiding the co-amplification of invertebrate DNA (BCV-RV1 5′-GCY CAN AYY ATN CYY RTR (T)(A)-3′). Three nucleotide positions always differing between vertebrate and invertebrate COI sequences are underlined. An additional difference in base composition, which position varies depending on particular arthropod Families, is indicated in brackets. A positional nucleotide numerical summary carried out using BioEdit revealed that the matching of our forward primer to the target sequence was >99% at each nucleotide position, after analyzing more than 6,000 COI sequences. The vertebrate-specific reverse primer matched the target sequence with values >99% at every nucleotide position, after comparing a minimum number of 5,814 vertebrate COI sequences from the four taxonomic Classes. The mismatching of invertebrate COI sequences with respect to our vertebrate-specific primer was also >99% in at least all the four nucleotide positions mentioned above. This estimate was calculated after comparing 3,273 arthropod COI sequences.

### PCR amplification and sequencing of vertebrate COI sequences

The expected length (excluding primers) of the PCR product obtained with primers M13BCV-FW and BCV-RV1 is 758 bp. An optimized PCR protocol consisted of 4 min at 94°C, followed by 35 cycles of 40 s at 45°C, 1 min at 72°C and 40 s at 94°C, with a final extension step of 7 min at 72°C. PCR reactions were carried out using a PTC-100 Programmable Thermal Controller (MJ Research) in a final volume of 30 µl containing 1 unit of a commercial Taq Polymerase (Bioline), 1X manufacturer-supplied buffer (Bioline), 2.5 mM MgCl_2_, 0.25 mM of each dNTP, 5% DMSO, 10 µg of BSA (Bovine Serum Albumin - Amersham corp.), 0.16 µM of primers M13BCV-FW and BCV-RV1 and 1 µl of extracted DNA. Bloodmeals can be sometimes partially digested, and thus, the number of DNA molecules to be used as templates decreases. Gradual digestion of DNA in arthropod's midgut should be heterogeneous across the sample, and depends on the time elapsed since the ectoparasite has fed. Thus, in order to standardize the number of amplified DNA copies before sequencing, we designed a PCR re-amplification protocol. Further, re-amplification of apparently negative PCR reactions has proven to be successful in previous studies [17]. In this second PCR reaction, we used the M13 primer and a modified reverse primer from that of the first PCR (BCV-RV2 = 5′-GCY CAN AYY ATN CYY RTR TAN CC-3′). Terminal cytosine nucleotides match to two conserved guanines of the COI locus in eukaryotes. This second PCR reaction consisted of 3 min at 94°C followed by a touch down protocol decreasing the annealing temperature from 60°C to 45°C during 40 s (−1°C/cycle), with 1 min extension and 40 s denaturalization steps at 72°C and 94°C, respectively, followed by 24 cycles of 40 s at 45°C, 72°C and 94°C, and a final elongation step of 7 min at 72°C. PCR reactions were carried out in a final volume of 30 µl containing 1 unit of Taq Polymerase (Bioline), 1X manufacturer-supplied buffer, 1.7 mM MgCl_2_, 0.25 mM of each dNTP, 5% DMSO, 5 µg of BSA, 0.16 µM of M13 and BCV-RV2 primers and 1 µl of the PCR products obtained during the first amplification step (M13BC-FW/BCV-RV1). We used negative controls of PCR amplification (i.e. absence of DNA template) to detect contaminations derived from the presence of bovine (because of the use of BSA) and human DNA. As positive control, we used the bloodmeal of a mosquito sampled while feeding on a mallard duck captured for ringing. A further analysis of repeatability was accomplished by applying the same protocol to a couple of bloodmeals reporting rare hosts (i.e. hosts only found in one bloodmeal). The species used for the repeatability analyses were the crested lark *Galerida cristata* and the Egyptian mongoose *Herpestes ichneumon*. The COI locus from both species was amplified and sequenced independently in three different occasions. An identical PCR protocol was applied to DNA extracts obtained from the abdomen of non-engorged mosquitoes (*Culex* sp., N = 2), ticks (*Hyalomma* sp., N = 2), culicoids (*Culicoides* sp., N = 2) and sand flies (*Phlebotomus* sp., N = 2) to ensure the unsuccessful amplification of invertebrate DNA. Vertebrate DNA was used during these PCR experiments to control for systematic PCR failure. To identify problems related to the quality of the invertebrate DNA extracts, we performed additional PCR experiments using the eukaryote-universal mini-barcoding primers proposed by Meusnier and co-workers [13]. PCR products were visualized in 1.5% agarose gels and cleaned-up using the commercial ExoSAP-IT reagent (GE Healthcare Life Sciences). Sequencing reactions were performed according to the BigDye 1.1 technology (Applied Biosystems) using 5 pmoles of the BCV-RV2 primer. This primer is located around 100 bp downstream from the 3′ end of the molecular target for DNA barcoding. Labelled DNA fragments were resolved using an ABI 3130xl automated sequencer (Applied Biosystems).

### Identifying unambiguous and ambiguous unknown COI sequences

We used the identification engine implemented in the BOLD-IDS platform (http://www.barcodinglife.org/views/idrequest.php) to assign unknown COI sequences to particular species. The BOLD-IDS component provides a species identification tool that accepts DNA sequences from the barcode region and returns a taxonomic assignment to the species level when possible. We always chose to search within the reference barcode dataset (i.e. validated subset of the full database with a minimum sequence length of 500 bp and containing only those species represented by three or more individuals showing less than 2% sequence divergence) when possible.

Additionally, haematophagous arthropods can feed on several hosts. If hosts belong to different species, the resulting sequencing electropherogram will contain a variable number of ambiguities (i.e. double peaks). We used BioEdit to introduce IUPAC degenerate codes (i.e. those representing different sets of nucleotides) across sequencing electropherograms. Ambiguous COI sequences were aligned with respect to a reference data set containing unambiguous COI sequences from the vertebrate species identified in our study area so far. Then, we removed from the alignment those COI sequences differing from the ambiguous unknown sequence in at least 2% of nucleotide positions (i.e. the within-species threshold proposed by the DNA barcoding initiative) until only a few candidate species remained.
