# Correction: Disentangling Vector-Borne Transmission Networks: A Universal DNA Barcoding Method to Identify Vertebrate Hosts from Arthropod Bloodmeals

**DOI:** 10.1371/annotation/ce5bff53-4638-4931-aa68-904b74db4b20

**Published:** 2010-10-18

**Authors:** Miguel Alcaide, Ciro Rico, Santiago Ruiz, Ramón Soriguer, Joaquín Muñoz, Jordi Figuerola

In the Materials and Methods section two optimal primer sequences were switched by mistake with sub-optimal primer sequences. The correct sentences should be:

"In this second PCR reaction, we used an M13 Primer (GTA AAA CGA CGG CCA GTG) and a modified reverse primer from that of the first PCR (BCV-RV2=5' ACY ATN CCY ATR TAN CCR AAN GG-3'." The sentence "Terminal cytosine nucleotides match to two conserved guanines of the COI locus in eukaryotes" should be omitted.

