# Correction: Disentangling Vector-Borne Transmission Networks: A Universal DNA Barcoding Method to Identify Vertebrate Hosts from Arthropod Bloodmeals

**DOI:** 10.1371/annotation/1e67b1e0-a356-4b3a-8c4c-03037ccfe1f7

**Published:** 2010-10-18

**Authors:** Miguel Alcaide, Ciro Rico, Santiago Ruiz, Ramón Soriguer, Joaquín Muñoz, Jordi Figuerola

In the Materials and Methods section two optimal primer sequences were switched by mistake with sub-optimal primer sequences. The correct sentences should be:

"We designed a universal-forward primer with an M13-tail added at the 5' end (M13BC-FW 5'-TGT AAA ACG ACG GCC AGT-HAA YCA YAA RGA YAT YGG -3'), similar to other primers previously used in other barcode approaches [e.g. 13]. Then, we searched for a reverse primer that allowed the specific amplification of vertebrate COI sequences while avoiding the co-amplification of invertebrate DNA (BCV-RV1 59-GCY CAN ACY ATN CCY ATR (T)(A)-3')."

